# Improving Efficiency of Multidisciplinary Bedside Rounds in the NICU: A Single Centre QI Project

**DOI:** 10.1097/pq9.0000000000000511

**Published:** 2022-01-21

**Authors:** Sandesh Shivananda, Horacio Osiovich, Julie de Salaberry, Valoria Hait, Kanekal S. Gautham

**Affiliations:** From the * University of British Columbia; † Division of Neonatology, BC Women’s Hospital and Health Centre; ‡ Department of Pediatric Medicine, Section of Neonatology, Houston, Tex.; § Baylor College of Medicine Houston, Tex.

## Abstract

**Introduction:**

Inconsistent workflow, communication, and role clarity generate inefficiencies during bedside rounds in a neonatal intensive care unit. These inefficiencies compromise the time needed for essential activities and result in reduced staff and family satisfaction. This study’s primary aim was to reduce the mean duration of bedside rounds by 25% within 3 months by redesigning the rounding processes and applying QI principles. The secondary aims were to improve staff and family experience.

**Methods:**

We conducted this work in an academic 50-bed neonatal intensive care unit involving 350 staff members. The change interventions included: (i) reinforcing essential value-added activities like standardizing rounding time, the sequencing of patients rounded, sequencing each team member rounding presentations, team preparation, bedside presentation content, and time management; (ii) reducing non-value-added activities; and (iii) moving value-added nonessential activities outside of the rounds.

**Results:**

The mean duration of rounds decreased from 229 minutes in the pre-implementation to 132 minutes in the postimplementation phase. The proportion of staff showing satisfaction regarding various components of the rounds increased from 5% to 60%, and perceived staff involvement during the rounds increased from 70% to 77%. Ninety-three percent of family experience survey respondents expressed satisfaction at being invited for bedside reporting and being involved in decision-making or care planning. The staff did not report any adverse events related to the new rounds process.

**Conclusion:**

Redesigning bedside rounds improved staff engagement and workflow, resulting in efficient rounds and better staff experience.

## Abstract 2

Supplemental Digital Content is available in the text.

## INTRODUCTION

During bedside rounds in the neonatal intensive care unit (NICU), team members from multiple disciplines review information regarding the patient, decide about patient management, and communicate with the patient’s family.^1–5^ Such rounds can be nurse-driven or resident-driven.^6–8^ Ideally, bedside rounds should be efficient, satisfy the needs of all the stakeholders, and result in optimal care plans, with the patient’s family contributing to the plan.^5,9^ Unfortunately, clinical teams often face challenges in accomplishing all these goals simultaneously during rounds.^10–12^

### Problem Description

For several years, staff and families in our NICU have raised concerns that bedside rounds are inefficient, inconsistent, including variation in start time, duration, sequence of rounding and reporting, preparation, staff and families involvement, and teaching. In addition, NICU staff reported that during rounds, they experienced fatigue, could not concentrate, often missed breaks, and sometimes missed recognizing worsening patient acuity.^13^ After lengthy rounds, they often lacked time to implement plans.

In 2016, our hospital planned to expand our 16,000 sq ft open bay NICU, where patients were geographically grouped based on acuity, to an 80,000 sq ft single-room NICU without geographic acuity cohorting. In January 2017, 10 months before the planned move, we launched a quality improvement project with the primary aim of reducing the mean duration of bedside rounds by 25% within 3 months by redesigning the rounding process. The secondary aim was to improve staff and parent satisfaction with NICU rounds.

## METHODS

### Setting

We conducted the project in our 50-bed Level 4 NICU with 700 annual admissions, including inborn and outborn infants with medical and surgical conditions. The average daily NICU census is 43, with a mean Score for Neonatal Acute Physiology with Perinatal extension-II (SNAPPEII) of 16.^14,15^

### Baseline Process of NICU Rounds

Before the QI project initiation, night-shift staff performed only a few preparatory rounding activities, such as partially completing orders for parenteral nutrition and enteral feeding. The day-shift staff performed the bulk of rounding pre-work shortly after the morning patient hand-off. Two teams conducted NICU rounds concurrently. Each team, led by a neonatologist, included two to four house staff, a pharmacist, and a charge nurse. This team would move from bed to bed and interact with each patient’s nurse, a respiratory therapist (RT), and the patient’s parents. A dietitian was a part of each team on alternate days. Other NICU health professionals took part in rounds as needed. Each team started rounds by first discussing patients admitted overnight and those who were unstable.

### QI Project

Our QI team comprised a physician, a nurse quality lead, a nurse educator, a charge nurse, a family advisor, and a project facilitator. We developed a project charter, created process maps, deconstructed the problem using a cause-and-effect diagram, and performed a workflow analysis. We conducted the project in four phases: pre-implementation (Jan 1–Feb 21), implementation (Feb 22–28), post-implementation (Mar 1–Apr 30), and sustainment (May 1–June 12). We prespecified the dates of the first three phases to simplify scheduling for project team members.

### Design and Testing of Changes to Rounds

**Fig. 1.**
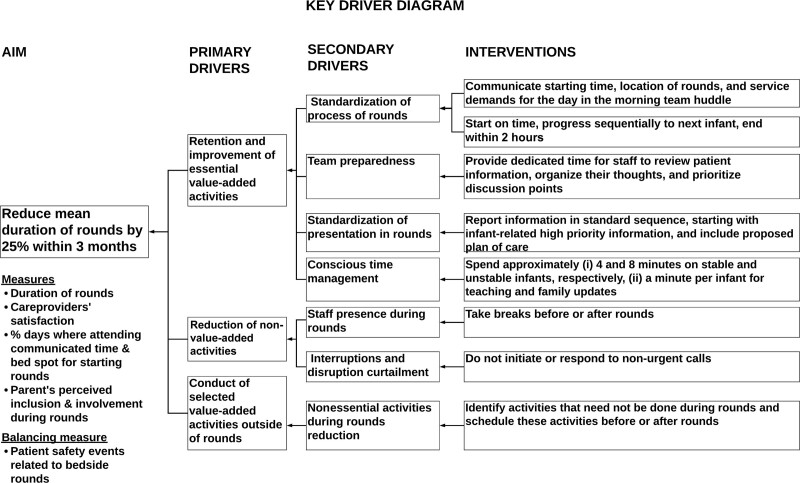
Key driver diagram for increasing rounds efficiency.

We hypothesized that the key strategies to shorten rounds and improve staff experience would be to incorporate activities that added benefit, built ownership among staff and families, and streamlined workflow. A literature review identified potential interventions to improve rounds and their implementation.^7,16,17^ We simulated a nurse-driven, resident-driven, and a combined interprofessional rounding model before agreeing on the combined model as it promoted ownership among all members. Then we created a key driver diagram with change ideas (Fig. 1).

After iterative discussions with broader NICU staff representatives, the team revised the initial change ideas to reflect a consensus set of implementable interventions that might achieve a feasible 25% reduction in rounds duration. The staff members agreed on a default time for rounds (09:45–11:45 am), a preparation period (08:30–09:45 am), and using the preparation period for discussing exceptionally sick infants.

**Fig. 2.**
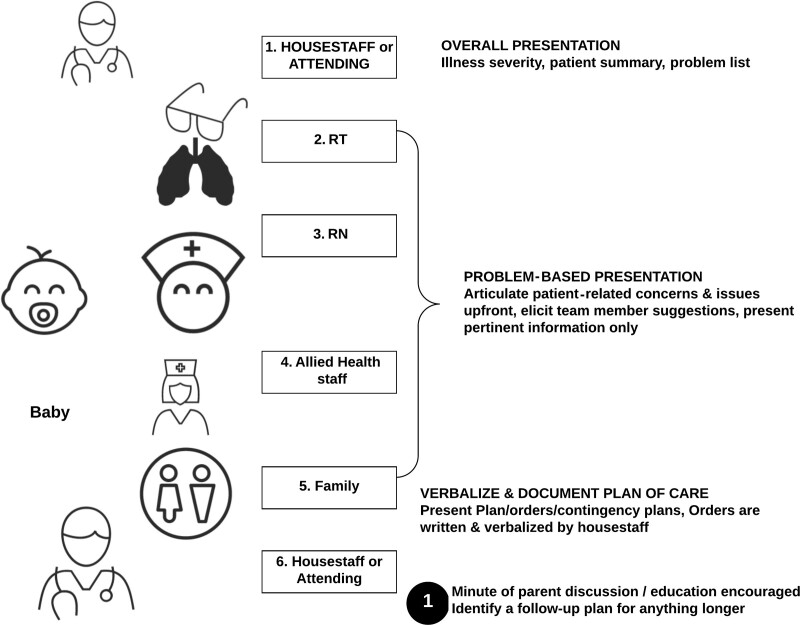
Bedside rounds presentation sequence by various team members. RN, Registered nurs; RT, Registered respiratory therapist.

The main eight interventions were: (i) communicating starting time and location of rounds, (ii) starting on time, progressing sequentially to the next infant, and ending within two hours, (iii) providing dedicated time for staff to review patient information, organize their thoughts, and prioritize discussion points, (iv) reporting information in a standard sequence (Fig. 2), starting with infant-related concerns, and including a proposed care plan, and (v) presenting only relevant and high priority information within the allocated time (**See document 1, Supplemental Digital Content 1,** which shows Bedside rounds nurses presentation template. NEWS, Neonatal Early Warning Signs; CNS, Central nervous system; GU, Genitourinary system; CVS, Cardiovascular system; ID, Infectious diseases; GI, Gastrointestinal system; BIIP, Behavioral Indicators of Infant Pain, TFI, Total Fluid Intake. http://links.lww.com/PQ9/A347), (vi) taking breaks before or after rounds, (vii) not starting or responding to nonurgent calls, and (viii) identifying extraneous activities for completion before or after rounds.

The QI team members communicated the planned changes via several formats (email, newsletter, pocket cards) and forums (staff education days, Edu-quicks—ie, 5- to 15-minute bedside conversation between QI team member and staff). All frontline staff received training on using templates supporting the redesigned rounds (**See document 1, Supplemental Digital Content 1,** which shows bedside rounds nurses presentation template. NEWS, Neonatal Early Warning Signs; CNS, Central nervous system; GU, Genitourinary system; CVS, Cardiovascular system; ID, Infectious diseases; GI, Gastrointestinal system; BIIP Behavioral Indicators of Infant Pain, TFI-Total Fluid Intake. http://links.lww.com/PQ9/A347) and bedside coaching. The charge nurses plotted daily team rounding duration on a poster board throughout the project. Three QI team members spearheaded the change management, acting as the point of care expert, coach, data collectors, and change adoption facilitators. At least one of them was available for 4 hours on each weekday.

The QI team met every 2 weeks to review identified gaps, develop corrective actions, and address barriers. On two occasions (postimplementation and sustainability phases), the team met with sponsors to assess implementation effectiveness and identify actions to increase sustainability using National Health Service sustainability tool.^18^ The team’s purpose was to achieve a score of 55 or higher (maximum of 100), as lower scores correlate with a low likelihood of sustainability.^18^ The QI team introduced a standard work document to coordinate communication events in NICU and a tool for families (**See document 2, Supplemental Digital Content 2,** which shows bedside rounds families presentation prompt, http://links.lww.com/PQ9/A348) to briefly frame and verbalize their concerns during rounds during the postimplementation phase. Other guidelines and procedures related to patient admission, flow, or discharge did not change during the project.

### Study of the Intervention, Measures, and Outcomes

The primary outcomes were (i) duration of daily rounds in minutes, defined as the time between commencement of discussion on the first patient by the team, and the end of the discussion on the last patient seen by the team,^10,16,17^ and (ii) average rounding time per patient calculated by dividing total duration of rounds by total patients in NICU on each day. The secondary outcome measures were staff and parents’ satisfaction with rounds. We measured the former using a survey tool^10^ administered during the pre-implementation and sustainment phases, and later using a shorter version of the survey tool during the sustainment phase. The process measures to assess compliance with change interventions were: (i) articulation of start time and bed spot where rounds begin, defined as a proportion of days when a neonatologist explicitly stated starting time and rounding location at the morning team huddle. QI facilitators gathered these data on 10 random days each during the postimplementation and sustainment phases; (ii) subjective assessment of start time, sequential progression, and reporting in a standard sequence by QI facilitators. We did not measure the actual time spent per patient, absence of staff during rounds because of breaks, adequacy of preparation, interruptions, and deferrals of value-added activities outside rounds. The balancing measure was patient safety events associated with bedside rounds, captured from the existing reporting system, and adjudicated by the quality assurance lead. The implementation measures were: (i) the number of staff education sessions and their attendance; (ii) sustainability scores in postimplementation and sustainability phases.^18^

### Analysis

To calculate the daily duration of rounds, we added the duration of rounds for each rounding team and divided by two. We calculated the mean of the daily duration of rounds for each project phase using data from every day in that phase. Finally, we analyzed the change in mean “daily duration of rounds” and “duration of rounds per patient per day” over time using X bar control charts with standard rules for identifying special cause variation (QI Macros for excel 2018, KnowWare International, Inc., Denver, Colo.).^19–22^ We compared the proportion of respondents who “agreed or strongly agreed” with survey questionnaire statements before and after change using Chi-square or Fischer exact test.

### Ethical Considerations

Based on an initial assessment showing minimal risk for this project,^23^ the QI team did not apply for a full ethics board review, but adhered to the hospital’s information, risk, and privacy policies.

## RESULTS

### Demographics

During the study period, we admitted 230 infants to NICU with a mean ± standard deviation (SD), length of stay of 24 ± 12 days, gestation of 34 ± 5 weeks, and a birth weight of 2379 ± 1036 g. We collected rounds data on 120 days and on 262 infants. Twelve neonatologists, 22 house staff, 250 nurses, 35 respiratory therapists, and 20 allied staff members took part in the rounds that yielded the project data.

### Roll out

All the predetermined interventions described under change concepts for improvement in the key driver diagram (Fig. 1) were implemented in the third week of Feb 2017. Every day the QI facilitators briefed the team at the beginning of rounds, explaining the goals and time allocation limits of redesigned rounds, reminded neonatologists when they exceeded time limits per patient, and debriefed at the end of rounds (**See document 3, Supplemental Digital Content 3,** which shows bedside rounds time management and observational tool. RN, registered nurse; RT, registered respiratory therapist; MRP, most responsible physician. http://links.lww.com/PQ9/A349).

### Duration of Rounds

**Fig. 3.**
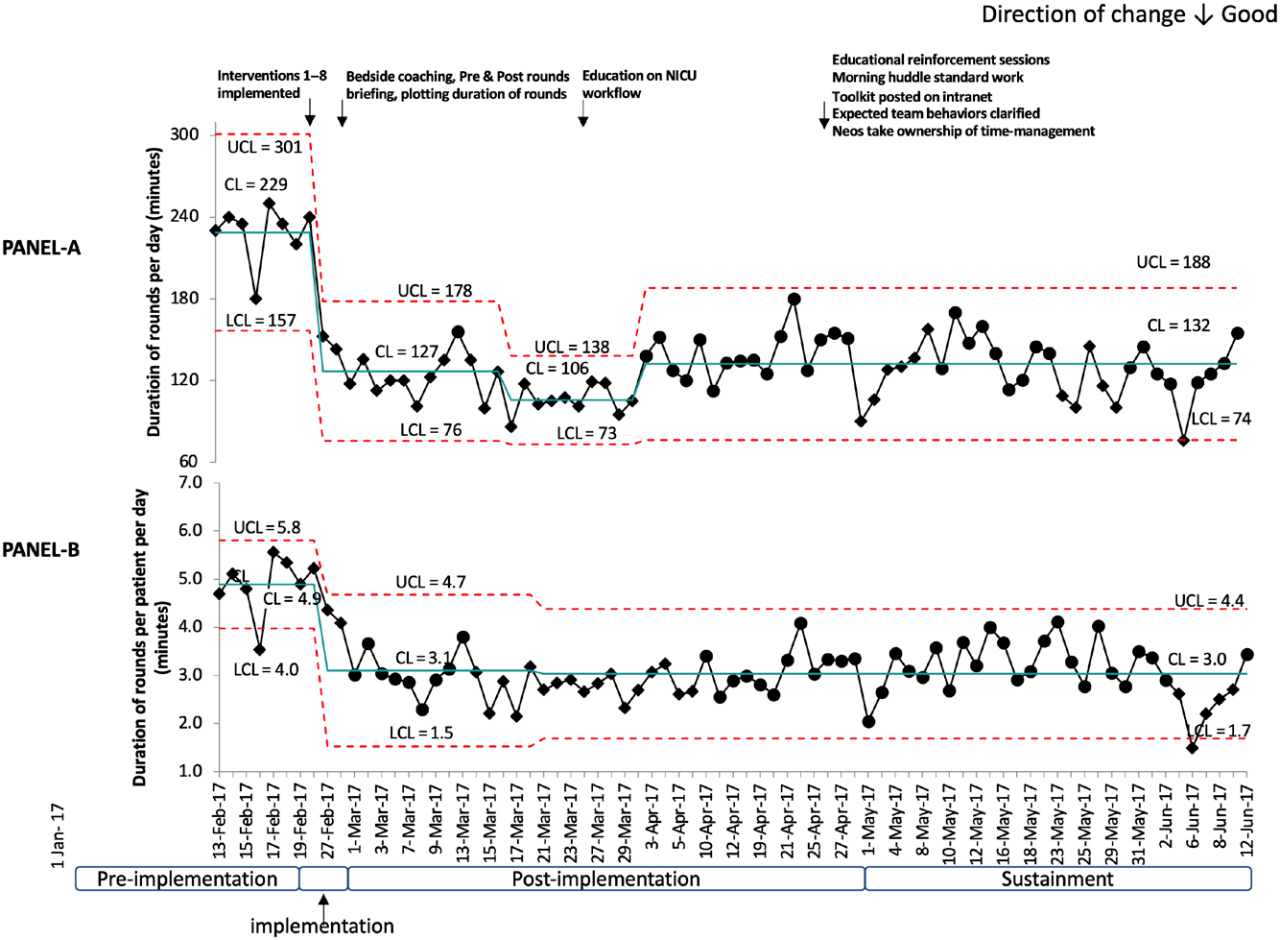
Control charts showing the duration of rounds per day and duration of rounds per patient per day during the study. Annotated X bar control charts. Each dot represents a daily rounding time (A) and rounding time per patient per day (B); the central line shows the mean duration of rounds (A) and mean duration of rounds per patient (B). Diamond- and circle-shaped dots represent unstable points (out-of-control process) and stable points, respectively. An intervention or an event followed by a run of eight points below the previous centreline was used to determine the centreline shifts. Interventions 1–8 implemented were: 1. Communicating starting time and location; 2. Starting on time and completing within 2 hours; 3. Creating a dedicated time to prepare for rounds; 4. Reporting information in a standard sequence; 5. Presenting only relevant information within the allocated time; 6. Organizing staff breaks outside rounding time; 7. Encouraging staff not to start or respond to nonurgent calls; 8. Identifying activities that need not be done during rounds and scheduling them before or after rounds. One data point (Feb 16) was excluded (ghosting) from calculating the initial centreline as the rounding teams expedited rounds due to scheduled neonatal mortality review meeting, a special cause.

The mean duration of rounds decreased from 229 minutes in the pre-implementation to 132 minutes in the post-implementation phase (Fig. 3). Following change interventions, the daily rounds duration and duration of rounds per patient showed significant and sustained reduction that continued for several weeks to months. Special cause variation with centre line shifts (Fig. 3) was noted during the implementation and postimplementation phases beginning on Feb 27 and Mar 17 for the rounding duration (likely a direct effect of implementing eight predetermined interventions), and Feb 27 and Mar 21 for the duration of rounds per patient (likely because staff become accustomed to the new processes). There were a few single-point special cause variation signals of unusually short (Feb 16th and Jun 6th) or unusually long (Apr 3rd and May 1st) rounds on the control charts (Fig. 3). The QI team investigated special causes causing these signals. Sometimes, team members had shortened rounds because they had to attend meetings. In other cases, the teams paused rounds to help with the admission of a critically ill patient, deal with a high census, or other reasons. When the sustainability score decreased to 45 during 1 week in April (3rd–10th), corrective actions were taken, which included educating team members, setting clear expectations, clarifying roles, and providing easy access to tools and template for rounds. In response, the mean durations of rounds stabilized, and the sustainability score increased to 54 (June 6th–12th).

### Staff Satisfaction and Family Experience

During the pre-implementation (Jan 1–Feb 21) and sustainment periods (May 1–June 12) of the project, 108 staff (51% of staff working in the unit that week) and 38 staff (19% of staff working in the unit that week) respectively responded to the survey. Nurses accounted for 75% and 68% of respondents in the pre- and post-implementation survey. The new rounding process’s satisfaction scores improved by 5%–60% on various components of rounds (**See document 4, Supplemental Digital Content 4,** which shows NICU staff satisfaction survey results with redesigned rounding process. Statements 1 and 2 show efficiency of rounds; statements 3, 4, 5, & 6 show a perceived impact of achieving goals of bedside rounds; statements 7, 8, & 9 show a perceived impact of rounds on staff engagement; statements 10, 11, & 12 show caregivers’ perception on preparation for rounds; statement 13 shows standardization of rounds presentation using a template; statements 14, 15 & 16 show time allocation; statement 17 shows reducing nonvalue-added activities. *P < 0.05 on Chi-square or Fischer exact test. Number of respondents in pre-implementation and sustainment phases were 108 and 38, respectively. http://links.lww.com/PQ9/A350). Similarly, the proportion of respondents who perceived “rounds delay patient-related priority activities” (survey question 6) decreased by 41% (*P* = 0.002).

Twenty parents, who made up 25% of parents of infants cared for in the NICU during the last week of the sustainment phase (June 6–12), responded to the family experience survey. All respondents indicated that the start time of rounds was predictable, and 93% of respondents expressed satisfaction at being invited for bedside reporting and decision-making or care planning involvement. We did not have comparative preintervention evaluation.

### Process Measures

The proportion of days where neonatologists articulated the start time and location of rounds increased from 70% in the post-implementation phase (Mar 1–Apr 30) to 90% in the sustainment phase (May 1–June 12).

### Balancing Measure

There were no rounds-related patient safety incidents or adverse events reported by staff in the pre-implementation or the subsequent phases.

### Implementation Measures

Two hundred and fifty-four staff took part in staff education days (Feb 1–8), 158 in the initial Edu-quick session (Feb 22–28, 88% of scheduled staff), and 73 in reinforcement Edu-quick sessions (May 1–May 14, 39% of scheduled staff). Twenty members, as well as NICU charge nurses, charge RTs, and flow coordinators received 2-hour coaching on coordinating all daily communication events, using a standard work document (Mar 1–Apr 30). The sustainability scores increased from 45 in the post-implementation phase to 54 in the sustainability phase. We share the mitigation strategies to address challenges during implementation in Table 1.

**Table 1.** Challenges and Mitigation Strategies during the Rounds Redesign Implementation

Challenges and Barriers	Mitigation Strategies
Disconnect among team members in understanding patient acuity status	QI team encouraged staff to use standard terminology to describe patient acuity: stable, watcher, unstable, and critical
Continuing rounds immediately after x-ray rounds without taking time for preparation	Staff were requested to perform a set of activities to facilitate their preparation for rounds in the time between x-ray rounds and set starting time. It included the list of preparatory activities that could be done (learners training toolkit)
Inconsistency in articulating start time and location in the morning team huddle by neonatologists	A standard work document clarified everyone’s role and empowered charge nurses to seek this information from the attending neonatologist during huddle or by paging them before 8:30 am
Rigid standardized communication at bedside led to excessive time being spent on stable infants and inadequate time for unstable infants	Attending neonatologist took the responsibility for managing the 2 hours of rounding time between stable and unstable infants and meeting educational needs of learners and family updates
Team members not discussing expected problems and contingency plans to reduce length of rounds	QI team recommended rounding teams to have a contingency plan in a subgroup of infants who are unstable or critical
Inadequate and inconsistent written handover of "to-do" list from day to evening team members resulting in pending tasks for the next day’s team	House staff created and adopted a structured handover template
Lack of consensus on rounds process on weekends and weeknights.	QI team communicated the inability of medical staff members to stick to a consistent start time, and a neonatologist rounding on all infants because of low staffing level and unpredictable priorities
Charge nurses read out patient information from flow sheets when a bedside staff was on a break and managed the sequence of rounds resulting in inadequate ownership from bedside staff and attending neonatologists to take ownership of rounds.	Neonatal program medical and operational directors endorsed the attending neonatologists taking on the responsibility for leading and managing time during rounds. Charge nurses took ownership of planning and ensuring bedside staff were available consistently for rounds
Predictable rigid starting time of rounds prevented neonatologist from participating in subspecialty rounds or seeking input from subspecialists when they are in NICU during rounds	Neonatologist could briefly step out of rounds after delegating a member to lead rounds to ensure uninterrupted flow
Allocating time primarily for arriving at a daily plan of care and curtailing detailed teaching at bedside leading to frustration and a sense of loss among neonatologists	Attending neonatologist took the responsibility for managing the 2 hours of rounding time between stable and unstable infants, meeting educational needs of learners and family updates
To complete rounds within 2 hours, medical teams split into multiple physician teams. This often led to uni-disciplinary rounding, added confusion among team members and families, and occasionally resulting in repeating rounds with a neonatologist and changing daily plan of care	On creating awareness of drawbacks associated with splitting of rounds, the attending neonatologists and medical staff agreed on avoiding splitting of rounds
Families feeling rushed and all their questions were unaddressed during rounds	Bedside nurses-oriented families on the purpose of family interaction during rounds and encouraged families to request a time outside the rounds for detailed updates
Team members directing anger and frustration at another member during rounds redesign process	QI team along created a code of conduct document highlighting respectful behavior, inclusiveness, and safe environment while point of care staff were getting adapted to changes

NICU, neonatal intensive care unit; RT, registered respiratory therapist; QI, quality improvement.

## DISCUSSION

We have shown a significant sustained reduction in duration of rounds and higher staff satisfaction with no reported adverse events after redesigning the bedside rounding process in a large NICU. Implementing changes in bedside rounds and integrating them into the NICU routine was feasible in a complex setting.

A reduction in the duration of rounds coincided with implementing evidence-based interventions,^7^ complemented by robust change management. Therefore, we believe that the shortened rounding duration resulted from implementing changes and not from fluctuations in NICU patient census, patient acuity, or staffing of rounding teams. Because we implemented all eight interventions concurrently, we could not determine which individual intervention had the most significant impact on the rounding time. We speculate that dedicated preparation period, structured sequence of reporting, and attendings taking ownership during rounding may have had more impact than other interventions, as these interventions were not part of prior efforts to improve rounds in 2008 and 2013. Although not quantified and directly measured, our QI facilitators’ impression was that the rounding team members almost always followed through with their committed start time and location, used any available time before rounding for preparation, and rounds progressed sequentially to the next infant, while team members adhered to a standard sequence of presentation. Though desirable, we could not measure compliance of every intervention in the bundle because of limited facilitator availability and resentment by some care providers about scrutinizing activities such as going on breaks, time spent per patient, and time management. We believe team morale would have been significantly harmed if we had pursued that line of scrutiny.

We encountered two important challenges early in this project, deciding when and at which bedside location rounds would begin, and identifying which components of rounds would take priority over others. For the former, we achieved a consensus that the neonatologist, during the morning team huddle, would review various competing priorities such as the acuity of patients, overnight admissions, planned procedures, and discharges to make a decision. For the latter, the consensus was planning for care and that the neonatologist would manage time during rounds.

Interestingly, in this study there was a small increase in the proportion of staff feeling that the length of rounds had increased because of parent involvement, consistent with other studies.^24–26^ This observation should be balanced against the reported benefits of parental presence during rounds, such as building trust, understanding parents’ needs, providing emotional support, enhanced communication among rounding team, and integrating family input in careful decision making.^24,27–30^ Because none of the studies objectively measuring the duration of rounds with family participation have showed a significant increase in rounding time,^31–33^ the net benefit of parent-integrated rounds^27,34^ likely outweighs the perceived longer duration of rounds by staff in this study.

Our study’s results are consistent with previously reported quantitative and qualitative improvements in bedside rounds through interventions aimed at improving rounding practices,^10,35–37^ communication strategies,^16^ information exchange,^38^ and collaborative bedside decision making.^39^ We could not compare the magnitude of benefit observed in our study with above studies, as study setting, goals, co-interventions, design, and patient population varied widely. Vats et al described the impact of an intervention bundle on daily bedside rounds in PICU patients.^10^ Post intervention, their rounding time decreased by 23%, as opposed to 42% decrease observed in this study. Improvement in staff satisfaction on efficiency, timeliness, and reliability of rounding times (*P* < 0.05) was similar between both studies. A higher decrease in rounding time that was observed in this study could be secondary to comprehensive intervention bundle and its implementation, apart from differences in patient population and unit characteristics.

The strengths of the present study include the use of evidence-based interventions, customizing implementation strategies to suit the local needs, enabling staff to adopt new practices, measuring duration of rounds on all patient weekdays during the entire study, and use of control charts.^2,10,17,40–43^ The study approach is flexible and likely adoptable by another center.

Our work has several limitations. First, our baseline (pre-intervention period) has fewer data points (7 points) that is sub-optimal to establish a clear, stable baseline period. The baseline points were consistent with little variation, suggesting a reasonable baseline time-period. In addition, the pre-intervention baseline data occurred during some of the highest infant census periods and may have inflated the observed beneficial effect. However, there remained an impact on time spent per patient, which should be less sensitive to census. Second, changes introduced as a care bundle and not in a stepwise fashion prevent a clear estimation of which bundle elements had the greatest impact on the shortening of the rounds. Third, the anonymous survey data are subject to bias toward those who chose to respond. Further, the lower staff survey response rate during the sustainment phase could be secondary to staff fatigue with multiple site redevelopment-related concurrent surveys. Fourth, we limited the family experience survey to the post-implementation period, preventing evaluation of perceived experience changes. Fifth, as with many QI-related projects, resources can often present challenges and thus our work may not easily generalizable to other hospitals with different resource constraints. The key enablers of this project, such as codifying a quality improvement team and supporting change management, may be challenging for centers with limited resources.

## CONCLUSIONS

Redesigning the bedside rounding process by incorporating essential value-added activities, creating ownership, and aligning rounding practices to providers’ workflow significantly reduced the duration of rounds without compromising safety. Post-intervention, the proportion of staff reporting satisfaction and positive experiences increased.

## DISCLOSURE

The authors have no financial interest to declare in relation to the content of this article.

## ACKNOWLEDGMENTS

Help with the study: We thank all the neonatologists; Albersheim S, Kieran E, Lavoie P, Manhas D, Castaldo M, Panczuk J, Solimano A, Synnes A, Ting J, Wong J, for their input during the creation and implementation of change interventions. We are grateful to QI team members Hamman R, Leong B, Lekhi S, Harris K, Antrim A, Walker S, Abrams M, and Spindor N for being part of the QI team and taking part in change management. We appreciate English T and Esther C for editing the article. We thank all house staff, charge nurses, practice leaders, educators, managers, charge respiratory therapists for being engaged as early adopters and working through their change process, leading change process within their teams, and supporting reinforcement mechanisms, including audits, compliance measuring, ongoing training, and coaching. We thank all BCWH NICU, point-of-care staff, and senior executives for encouragement and support during implementation. We thank the neonatal program for seconding three experienced nurses as change facilitators by rotation for 4 months.

## Supplementary Material



## References

[1] Miller A Scheinkestel C Limpus A. Uni- and interdisciplinary effects on round and handover content in intensive care units. Hum Factors. 2009;51:339–353. 19750796 10.1177/0018720809338188

[2] Miller A Scheinkestel C Joseph M. Coordination and continuity of intensive care unit patient care. Hum Factors. 2009;51:354–367. 19750797 10.1177/0018720809340032

[3] Curley C McEachern JE Speroff T. A firm trial of interdisciplinary rounds on the inpatient medical wards: an intervention designed using continuous quality improvement. Med Care. 1998;36(8 Suppl):AS4–A12. 9708578 10.1097/00005650-199808001-00002

[4] O’Mahony S Mazur E Charney P. Use of multidisciplinary rounds to simultaneously improve quality outcomes, enhance resident education, and shorten length of stay. J Gen Intern Med. 2007;22:1073–1079. 17486384 10.1007/s11606-007-0225-1 PMC2305734

[5] Busby A Gilchrist B. The role of the nurse in the medical ward round. J Adv Nurs. 1992;17:339–346. 1573102 10.1111/j.1365-2648.1992.tb01912.x

[6] Marshall CD Fay ME Phillips B. Implementing a standardized nurse-driven rounding protocol in a trauma-surgical intensive care unit: a single institution experience. Cureus. 2018;10:e3422. 30546974 10.7759/cureus.3422 PMC6289560

[7] Lane D Ferri M Lemaire J. A systematic review of evidence-informed practices for patient care rounds in the ICU*. Crit Care Med. 2013;41:2015–2029. 23666096 10.1097/CCM.0b013e31828a435f

[8] Goldman-Yassen AE Strauss SB Vlismas PP. Face-to-face: resident-led radiology medicine rounds facilitate evidence-based processes for clinical decision support. Curr Probl Diagn Radiol. 2021;50:580–584. 32561151 10.1067/j.cpradiol.2020.05.005

[9] Anderson DC Todd SR. Designing for multidisciplinary rounding practices in the critical care setting. World Health Design. 2011;4:80–86.

[10] Vats A Goin KH Villarreal MC. The impact of a lean rounding process in a pediatric intensive care unit. Crit Care Med. 2012;40:608–617. 21983366 10.1097/CCM.0b013e318232e2fc

[11] Pronovost PJ Angus DC Dorman T. Physician staffing patterns and clinical outcomes in critically ill patients: a systematic review. JAMA. 2002;288:2151–2162. 12413375 10.1001/jama.288.17.2151

[12] Durbin CG Jr. Team model: advocating for the optimal method of care delivery in the intensive care unit. Crit Care Med. 2006;34(3 Suppl):12. 10.1097/01.CCM.0000199985.72497.D1 16477198

[13] Wetzel EA Lang TR Pendergrass TL. Identification of latent safety threats using high-fidelity simulation-based training with multidisciplinary neonatology teams. Jt Comm J Qual Patient Saf. 2013;39:268–273. 23789165 10.1016/s1553-7250(13)39037-0

[14] Shah PS Lee SK Yoon W. The Canadian Neonatal Net- work annual report. 2016. Available at http://www.canadianneonatalnetwork.org/Portal/LinkClick.aspx?fileticket=9K3crPtfgQs%3d&tabid=39. Accessed May 28, 2020.

[15] Shah PS Lee SK Yoon W. The Canadian Neonatal Net- work annual report. 2015. Available at http://www.canadianneonatalnetwork.org/Portal/LinkClick.aspx?fileticket=9K3crPtfgQs%3d&tabid=39. Accessed 28 May, 2020.

[16] Dodek PM Raboud J. Explicit approach to rounds in an ICU improves communication and satisfaction of providers. Intensive Care Med. 2003;29:1584–1588. 12898001 10.1007/s00134-003-1815-y

[17] Sharma S Hashmi MF Friede R. Interprofessional Rounds in the ICU. Treasure Island, Fla.: StatPearls Publishing LLC; 2020. 29939553

[18] Maher L Gustafson D Evans A. NHS Sustainability Model and Guide. University of Warwick; 2010.

[19] Shewhart WA, ed. Economic Control of Quality of Manufactured Product. D. Van Nostrand Company, Inc; 1931.

[20] Shewhart WA Deming WE, eds. Statistical Method from the Viewpoint of Quality Control. The Graduate school, the Department of agriculture; 1939.

[21] Wheeler DJ. Understanding Variation: The Key to Managing Chaos. SPC Press; 1993.

[22] Provost LP Murray S. The Health Care Data Guide: Learning from Data for Improvement. Jossey-Bass; 2011.

[23] Alberta Innovates. ARECCI ethics guideline tool. Available at albertainnovates.ca/wp-content/uploads/2017/11/ARECCI-Ethics-Guideline-Tool.pdf. Accessed May 28, 2020.

[24] Muething SE Kotagal UR Schoettker PJ. Family-centered bedside rounds: a new approach to patient care and teaching. Pediatrics. 2007;119:829–832. 17403858 10.1542/peds.2006-2528

[25] Grzyb MJ Coo H Rühland L. Views of parents and health-care providers regarding parental presence at bedside rounds in a neonatal intensive care unit. J Perinatol. 2014;34:143–148. 24202296 10.1038/jp.2013.144

[26] McPherson G Jefferson R Kissoon N. Toward the inclusion of parents on pediatric critical care unit rounds. Pediatr Crit Care Med. 2011;12:e255–e261. 21057363 10.1097/PCC.0b013e3181fe4266

[27] Seaman JB Arnold RM Scheunemann LP. An integrated framework for effective and efficient communication with families in the adult intensive care unit. Ann Am Thorac Soc. 2017;14:1015–1020. 28282227 10.1513/AnnalsATS.201612-965OI PMC5566311

[28] Brilli RJ Crandall WV Berry JC. A patient/family-centered strategic plan can drive significant improvement. Adv Pediatr. 2014;61:197–214. 25037128 10.1016/j.yapd.2014.03.009

[29] Kuo DZ Sisterhen LL Sigrest TE. Family experiences and pediatric health services use associated with family-centered rounds. Pediatrics. 2012;130:299–305. 22778299 10.1542/peds.2011-2623 PMC3408683

[30] Davidson JE. Family presence on rounds in neonatal, pediatric, and adult intensive care units. Ann Am Thorac Soc. 2013;10:152–156. 23607848 10.1513/AnnalsATS.201301-006PS

[31] Cameron MA Schleien CL Morris MC. Parental presence on pediatric intensive care unit rounds. J Pediatr. 2009;155:522–528. 19555968 10.1016/j.jpeds.2009.03.035

[32] Phipps LM Bartke CN Spear DA. Assessment of parental presence during bedside pediatric intensive care unit rounds: effect on duration, teaching, and privacy. Pediatr Crit Care Med. 2007;8:220–224. 17417129 10.1097/01.PCC.0000262798.84416.C5

[33] Ladak LA Premji SS Amanullah MM. Family-centered rounds in Pakistani pediatric intensive care settings: non-randomized pre- and post-study design. Int J Nurs Stud. 2013;50:717–726. 22704527 10.1016/j.ijnurstu.2012.05.009

[34] Harris GM. Family-centered rounds in the neonatal intensive care unit. Nurs Womens Health. 2014;18:18–27. 24548493 10.1111/1751-486X.12090

[35] Bradshaw KE Gardner RM Clemmer TP. Physician decision-making–evaluation of data used in a computerized ICU. Int J Clin Monit Comput. 1984;1:81–91. 6400423 10.1007/BF01872746

[36] Friesdorf W Konichezky S Gross-Alltag F. System ergonomic analysis of the morning ward round in an intensive care unit. J Clin Monit. 1994;10:201–209. 8027753 10.1007/BF02908862

[37] Cardarelli M Vaidya V Conway D. Dissecting multidisciplinary cardiac surgery rounds. Ann Thorac Surg. 2009;88:809–813. 19699903 10.1016/j.athoracsur.2009.05.007

[38] Collins SA Bakken S Vawdrey DK. Agreement between common goals discussed and documented in the ICU. J Am Med Inform Assoc. 2011;18:45–50. 21113075 10.1136/jamia.2010.006437 PMC3005873

[39] Hill K. The sound of silence–nurses’ non-verbal interaction within the ward round. Nurs Crit Care. 2003;8:231–239. 14725388 10.1111/j.1362-1017.2003.00038.x

[40] Harvey G Kitson A. PARIHS revisited: from heuristic to integrated framework for the successful implementation of knowledge into practice. Implement Sci. 2016;11:33. 27013464 10.1186/s13012-016-0398-2 PMC4807546

[41] Stevens B Lee SK Law MP; Canadian Neonatal Network EPIC Study Group. A qualitative examination of changing practice in Canadian neonatal intensive care units. J Eval Clin Pract. 2007;13:287–294. 17378877 10.1111/j.1365-2753.2006.00697.x

[42] ADKAR Model Of Change. Change management coach. Available at http://www.change-management-coach.com/adkar.html. Accessed 25 March, 2017.

[43] Davidson JE Savidan KA Barker N. Using evidence to overcome obstacles to family presence. Crit Care Nurs Q.2014;37:407–421. 25185768 10.1097/CNQ.0000000000000041

