# Supplementary material for: Improving Efficiency of Multidisciplinary Bedside Rounds in the NICU: A Single Centre QI Project
Source: Pediatr Qual Saf. 2022 Jan 21;7(1):e511. doi: 10.1097/pq9.0000000000000511 (PMC8782118; doi:10.1097/pq9.0000000000000511)
Supplement: Supplementary file 1 [file pqs-7-e511-s001.pdf]

ROUNDS PRESENTATION PROMPT

Prepare your presentation for rounds by following steps 1 through 3 below (MANDATORY PRESENTATION IN RED)

1. The Patient is ☐ Stable ☐ Watcher (Change in Status e.g. refer to NEWS) ☐ Unstable

2. Gestational/Corrected Age ___________ Patient Weight _______________ Gain/Loss____________

3. My priority concerns for this baby are:

**CNS**<br>**BIIP/response to handling**<br>**Current sedation**	**GU**<br>**Urine output**
CVS<br>Vascular/ arterial access	Medication<br>Current medications-
Respiratory<br>Additional concerns-	Labs<br>Bloodwork collected today-
Fluids and Nutrition<br>TFI/Fluid balance	ID/Skin
GI<br>Current feeds and frequency	Family/Discharge readiness<br>What needs to be done for transfer/discharge
