# Supplementary material for: Improving Efficiency of Multidisciplinary Bedside Rounds in the NICU: A Single Centre QI Project
Source: Pediatr Qual Saf. 2022 Jan 21;7(1):e511. doi: 10.1097/pq9.0000000000000511 (PMC8782118; doi:10.1097/pq9.0000000000000511)
Supplement: Supplementary file 2 [file pqs-7-e511-s002.pdf]

PARENT INTEGRATED ROUNDS

NICU Families Rounds Prompt Tool

ROUNDS TAKE PLACE BETWEEN 0945 to 1145 am

INSTRUCTIONS

- **Please discuss your concerns or observations with your baby's bedside nurse prior to the start of the rounds. This can be done**
  - **on the day or the evening before**
  - **over the phone prior to 0945 hrs**
  - **in-person, prior to the start of the rounds**
- **You are invited to present your concerns or observations about your baby during the rounds**
- **If all your questions or concerns cannot be covered during rounds, a plan will be made to follow-up with you later in the day.**
- **Please use this form as a guide to bring forward your concerns or questions about your baby to the rounds**

My top concerns for my baby are:

1 ______________________________

2 ______________________________

3 ______________________________

I have noticed ....

I would like to know/hear ....
