# Supplementary material for: Improving Efficiency of Multidisciplinary Bedside Rounds in the NICU: A Single Centre QI Project
Source: Pediatr Qual Saf. 2022 Jan 21;7(1):e511. doi: 10.1097/pq9.0000000000000511 (PMC8782118; doi:10.1097/pq9.0000000000000511)
Supplement: Supplementary file 3 [file pqs-7-e511-s003.pdf]

Time management and observation tool

<u>ROUNDS PROCESS</u>

- ❑ Overall Presentation
    - Preparation for rounds by all core team members (see preparation)
    - Initial presentation by MRP (May include head to toe presentation)
- ❑ Problem based presentation
    - Problem based presentation by RT first, followed by RN and facilitated by MRP (issue/concern supported by trends in data followed by a recommendation or request for recommendation)- Follow prompt
    - Problem based presentation by Allied Health ((issue/concern supported by trends in data followed by a recommendation)
    - Concerns/ issues from parents (if longer than a minute- identify a follow-up plan (Who will follow up and when?) with parent
- ❑ More than 1 minute teaching (learners)
    - Encourage teaching moments and learner concerns/ questions (if longer than a minute- identify a follow-up plan with the learner and / or take it outside of rounds). Accumulated questions/ issues by learner is to be discussed later after rounds
- ❑ More than 1 minute of parent discussion/education
    - Concerns/ issues from parents ((if longer than a minute- identify a follow-up plan (Who will follow up and when?) with parent
- ❑ Plan of care documented and verbalized
    - Plan/orders/contingency plan is to be presented by MRP/Delegate. Watcher and Unstable babies should have a contingency plan within their overall daily plan of care
    - Orders documented by house staff and read back to verify plans
- ❑ Overall time of rounds
- ❑ Target= 4minutes for stable infant and 8 minutes for unstable infants

RN-Registered nurse, RT- Registered respiratory therapist, MRP-Most responsible physician attending

<u>OBSERVATIONS</u>

(Please note any impact on family, staff, safety and efficiency)

Please bring these to the attention of the team members at the end of the rounds.

Please bring recurring themes to QI team meetings

Measures to collect and plot on the performance wall

❑ Start time of rounds:___. End time of rounds: ______

❑ If 0830 rounds occur: Start time: _____and End time: _____

❑ Did the team split: ____Yes or _____No
